# Modelling Chemotactic Motion of Cells in Biological Tissues

**DOI:** 10.1371/journal.pone.0165570

**Published:** 2016-10-31

**Authors:** Bakhtier Vasiev

**Affiliations:** Department of Mathematical Sciences, University of Liverpool, Liverpool, United Kingdom; Bioinformatics Institute, SINGAPORE

## Abstract

Developmental processes in biology are underlined by proliferation, differentiation and migration of cells. The latter two are interlinked since cellular differentiation is governed by the dynamics of morphogens which, in turn, is affected by the movement of cells. Mutual effects of morphogenetic and cell movement patterns are enhanced when the movement is due to chemotactic response of cells to the morphogens. In this study we introduce a mathematical model to analyse how this interplay can result in a steady movement of cells in a tissue and associated formation of travelling waves in a concentration field of morphogen. Using the model we have identified four chemotactic scenarios for migration of single cell or homogeneous group of cells in a tissue. Such a migration can take place if moving cells are (1) repelled by a chemical produced by themselves or (2) attracted by a chemical produced by the surrounding cells in a tissue. Furthermore, the group of cells can also move if cells in surrounding tissue are (3) repelled by a chemical produced by moving cells or (4) attracted by a chemical produced by surrounding cells themselves. The proposed mechanisms can underlie migration of cells during embryonic development as well as spread of metastatic cells.

## Introduction

**Fig 1:**
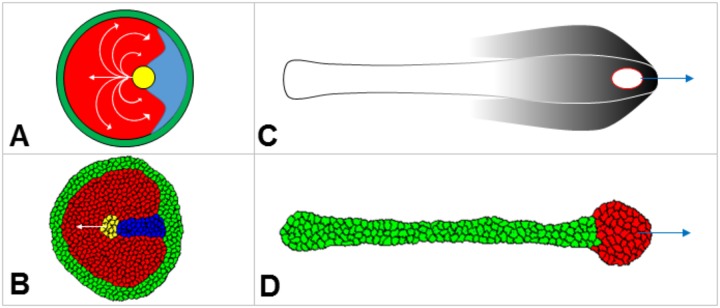
Migration of cells during gastrulation in the chick embryo. **A:** Schematic illustration of cellular flows in the chick embryo at early gastrulation [15]. Yellow spot represents a morphological structure called Hensen’s node which consists of roughly 80 cells (size is about 100μm) and moves with a speed of about 2μm/min. **B:** Process shown in panel **A** as modelled by Cellular Potts Model [8]. Translational motion of yellow cells can be explained by four chemotactic mechanisms: (i) yellow cells are attracted by chemical produced by red cells, (ii) yellow cells are repelled by chemical produced by blue cells, (iii) red cells are repelled by a chemical produced by yellow cells, and (iv) blue cells are attracted by a chemical produced by yellow cells. **C:** Schematic illustration of stem zone migration at late gastrulation in the chick embryo [16]. Red contoured white spot represents stem zone which consists of about 1000 cells (size is 0.3mm) and moves with a speed of about 2μm/min. **D:** Process shown in panel **C** as modelled by Cellular Potts Model [9]. Translational motion of the stem zone (represented by red cells which are proliferating and differentiating into green cells) is explained by chemo-repulsion by FGF8 [10] which is produced in the stem zone [17].

Mathematical analysis of morphogenetic patterns in developmental biology is commonly restricted by the case of stationary morphogen gradients [1–3]. The dynamical changes in concentration profiles, such as propagating waves and oscillations, are also considered but generally in a context of nonlinear interactions between morphogens [4]. It is known that the dynamics of morphogen gradients can also be affected by the movement of cells. Gastrulation in the chick embryo is a good example for which detailed data on the dynamics of gene expression patterns and cell movement is available [5–7]. Furthermore, the movement of cells can, in turn, be affected by morphogen concentrations, for example if the movement is chemotactic and morphogens act as chemotactic agents. These possibilities have recently been explored in studies of gastrulation combining mathematical modelling and experiments [8,9]. Early gastrulation in the chick embryo is associated with an extensive motion of cells which combines progressive (towards to future head) motion along the midline of bilaterally symmetric epiblast with vortex-type cellular flows on the both sides (Fig 1A). Simulations on the Cellular Potts Model have indicated that the motion along the midline can be due to chemotaxis if at least three cell types are involved in the movement scenario (Fig 1B) while vortices of cellular flows appear due to “viscous” interactions between chemotactically active and passive cells [8]. Late gastrulation is embarked by a movement of cells (forming so called “stem zone”) along the epiblast midline in the opposite direction (towards the future tail) (Fig 1C). Cells in the stem zone produce FGF8 protein which is known to act as chemorepellent [10]. Computer simulations on Cellular Potts Model have shown that the movement of the stem zone could be explained in an assumption that cells forming stem zone are repelled by FGF8 [9] (see Fig 1D).

Uncertainty with the modelling results in [8,9] comes from the fact that there is no direct experimental evidence confirming that the movement of cells in chick gastrula has chemotactic nature. However it is known that the cells which have undergone the epithelial-to-mesenchyme transition (EMT) chemotactically respond to FGF8 and FGF4 [10] and therefore could have this ability before the transition. Many researchers favour the cellular intercalation mechanism as an alternative for explanation of cell rearrangement during gastrulation [11]. Besides, the observation that the extracellular matrix in epiblast moves along with migrating cells (and has almost the same speed) [12] is considered as a strong argument against the chemotaxis since this brings up the problem of how the forces for chemotactic motion are exerted. The very same problem has been puzzling researchers in the case of moving *D*. *dictyostelium* slug [13] and there were suggested mechanisms for these forces to be translated from the interface between the slug and the substrate [14]. Similarly, forces exerted by cells moving chemotactically over the chick embryo epiblast can be translated from the epiblast periphery.

Models developed in [8,9] where overloaded by many details (concerning topology of the tissue, cellular differentiation and proliferation) to better describe particular biological processes while the proper analysis of mechanisms responsible for migration of cells was omitted in both cases. Such analysis would let the study made in [8] be extended to a theory of migration of nonhomogeneous group of chemotactically moving cells, while the study in [9]—to the theory of migration of homogeneous group. In this article we introduce a basic model (as well as a few of its variations) for the analysis of movement of homogeneous group of cells in biological tissue due to chemotaxis. In the model, presented here, we neglect “viscous” interactions between cells and consider movement of compact group of cells surrounded by immobile cells. We show that under broad range of conditions such movement can be explained by chemotactic response of moving cells (or even by such response of immobile surrounding cells) to chemical agents produced either inside or outside the moving group.

## Results

### Basic model: steady migration of self-repelling cells

**Fig 2:**
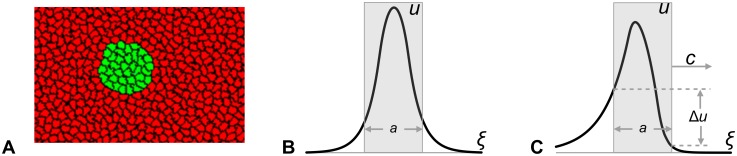
Illustrations of 2D- (A) and 1D- (B, C) models. **A:** Group of cells (green) forms a circular domain in a tissue (composed by red cells) in two-dimensional Cellular Potts Model [18]. **B-C**: One-dimensional model represented by a cross-section of two-dimensional model tissue (panel **A**) through the centre of the green domain. Group of cells now is represented by a segment of a line (of length *a*) along the *ξ*-axis. Symmetric *u*-profile for stationary (*c* = 0, panel **B**) and asymmetric *u*-profile for travelling wave (*c≠0*, panel **C**) solutions given by Eq (3) are shown. On the asymmetric profile (panel **C**) the maximum concentration lags behind the mid-point of the segment and the concentration on the back of the segment is above the concentration on its front (the difference is denoted by Δu on panel **C**).

Here we introduce a basic model for the analysis of movement of group of cells (or a single cell) in biological tissue due to chemotaxis. The developing tissue (i.e. epiblast of chick gastrula) is often represented by a unicellular layer of cells and therefore can be viewed as a two-dimensional object. Cellular movement patterns can be quite sophisticated [6], however, as a rule, there exists a compact group of cells (often referred to as an organising centre) whose movement orchestrates the movement of other cells in the tissue. In the basic model, presented here, we will neglect the mechanical interactions between cells and presume that there is a compact group of moving cells (which we will call the “moving domain” or MD) surrounded by immobile cells. In a two-dimensional model the MD can be viewed as forming a circular domain (Fig 2A). Here we design a one-dimensional model describing a cross-section of the planar system through the middle of the MD and parallel to the direction of its movement. Assuming that the shape (i.e. circular) of the MD is not changing we reduce its movement to the movement of a segment of line of constant length, *a*, along the line parallel to this segment and considered as *x*-axis (Fig 2B and 2C). Now, we assume that the cells forming the MD (the segment in 1D-model) produce a chemotactic agent which can diffuse into surrounding tissue and degrade. If the segment, where the chemical is produced, is moving then its concentration profile, *u(x*,*t)*, is dynamic and defined by the following initial value problem:

$$\frac{\partial u}{\partial t}=D\frac{\partial^{2}u}{\partial x^{2}}+p(x,t)-k_{1}u=0;\;u(x,0)=u_{0}(x).$$ (1)

Here the first term on the right-hand side describes the diffusion of chemical, the second—its production (inside the MD) and the third—the decay. Problem (1) should be stated on the domain (representing the tissue) with no flux boundary conditions. However to simplify the analytical solutions (to dismiss the effect of boundaries which is negligible for a sufficiently large domain) we consider the infinite medium. Furthermore, if the velocity of MD is constant then the Eq (1) can be rewritten in a frame of reference moving with the MD (i.e. using the substitution *ξ = x-ct* where *c* is a velocity of the MD) as the following:

$$\frac{\partial u}{\partial t}=D\frac{\partial^{2}u}{\partial \xi^{2}}+c\frac{\partial u}{\partial \xi }+p(\xi )-k_{1}u;\;u(\xi ,0)=u_{0}(\xi )\text{ where }p(\xi )=\{\begin{array}{l} k_{2}\text{ for }0\leq \xi \leq a\\ 0\text{ for }\xi <0\text{ and }\xi >a \end{array}.$$ (2)

The second term on the right-hand side of this equation takes into account the movement of domain (with velocity *c*) and function *p(ξ)* accounts for a constant-rate production of the chemical inside the domain (of length *a*). Stationary solution of Eq (2) (or so-called travelling wave solution) represents a conserved *u*-profile following the MD. Intuitively it is clear that whatever initial conditions we start from, if the speed of the MD is constant, then the *u*-profile should asymptotically become stationary (see S1 and S2 Movies). Formal analysis of Eq (2) indicates that the initial profile *u*_*0*_*(ξ)* transfers into the stationary solution with a relaxation time (for a slowest mode of transient solution) given by the reciprocal of the decay constant *k*_*1*_. The stationary *u*-profile (derived in an assumption that its first derivative is continuous on the borders of the domain and zero at infinity) is given by the following expression:

$$u_{st}(\xi ,c)=\{\begin{array}{ll} \frac{k_{2}}{k_{1}\left(\lambda_{1}-\lambda_{2}\right)}\lambda_{2}\left(e^{\lambda_{1}\left(\xi -a\right)}-e^{\lambda_{1}\xi }\right) & \text{for }\xi \leq 0;\\ \frac{k_{2}}{k_{1}\left(\lambda_{1}-\lambda_{2}\right)}\left(\lambda_{2}e^{\lambda_{1}\left(\xi -a\right)}-\lambda_{1}e^{\lambda_{2}\xi }+(\lambda_{1}-\lambda_{2})\right) & \text{for }0\leq \xi \leq a;\text{ where }\lambda_{1,2}=\frac{-c\pm \sqrt{c^{2}+4k_{1}D}}{2D}.\\ \frac{k_{2}}{k_{1}\left(\lambda_{1}-\lambda_{2}\right)}\lambda_{1}\left(e^{\lambda_{2}\left(\xi -a\right)\;}-e^{\lambda_{2}\xi }\right) & \text{for }\xi \geq a; \end{array}$$ (3)

The stationary concentration profiles of morphogen, *u*_*st*_*(ξ*,*c)*, for non-moving (*c = 0*) and moving to the right (*c>0*) domains are shown on panels B and C of Fig 2. The *u-*profile for the MD is asymmetric with the maximum concentration shifted to its back and the concentration on the front of the MD lower than on its back (the difference is denoted by *Δu* in Fig 2C). This concentration profile indicates that the movement of domain can be explained by a chemotaxis provided that the cells forming the MD are chemotactically repelled by the produced chemical. This statement brings together the observation that the *u*-level in front of the MD is lower compared to the *u*-level behind the MD, with the definition of chemo-repulsion as a motion towards an area of lower concentration. For mathematical description of chemotaxis we adopt (for now) the statement from the Keller-Segel model [19] that the speed of a chemotactically moving cell (treated as a mathematical point) is proportional to the gradient of chemotactic agent at its location, or *c = c*_*0*_*grad(u)*. Parameter *c*_*0*_ defines the balance between the force exerted by chemotactically moving cell and the resistance to this motion by the surrounding cells forming the tissue. Parameter *c*_*0*_ is positive for chemoattraction and negative for chemorepulsion. Since the chemotactically moving cells form a domain of finite size, the speed of the MD, *c*, is defined by the average gradient of the chemical, *u(ξ)*, over the MD, that is:

$$c=c_{0}\frac{u(a)-u(0)}{a}$$ (4)

which after substituting the expressions for *u(0)* and *u(a)*, for the stationary *u*-profile given by Eq (3), transforms into:

$$c=\frac{c_{0}k_{2}}{ak_{1}(\lambda_{1}-\lambda_{2})}\left(\lambda_{1}\left(1-e^{a\lambda_{2}}\right)+\lambda_{2}\left(1-e^{-a\lambda_{1}}\right)\right)\text{.}$$ (5)

Thus, we have a system where concentration *u(ξ)*, depends on speed *c* as given by Eq (3) and speed *c* is defined by the values *u(0)* and *u(a)* on the borders of the MD and therefore should satisfy the Eq (5). Eq (5) can be written as *c = f(c)* where *f(c)* is a short notation for the right-hand-side of Eq (5). Since *f(0) = 0* for all values of model parameters, the system has always solution represented by a stationary domain (*c = 0*).

**Fig 3:**
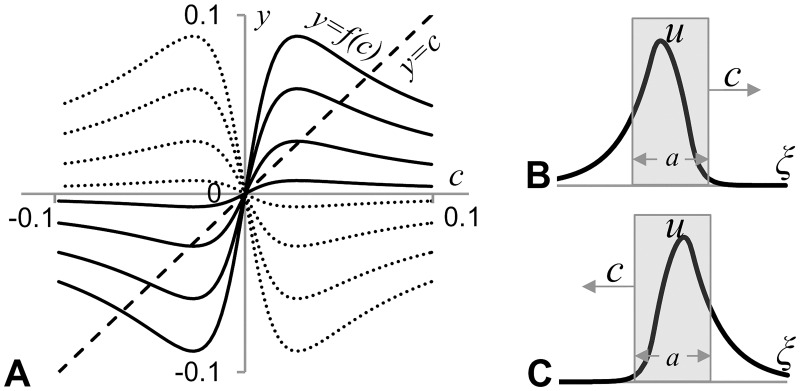
Travelling wave solutions in the system (2, 4). **A**: Plots of *y = f(c)* for four positive (dotted lines) and four negative (solid lines) values of *c*_*0*_ and the plot of *y = c* (dashed line) are shown. Abscissa of the points of intersection of solid/dotted lines with dashed one gives the speed, c, (for the value of *c*_*0*_ corresponding to the solid/dotted line) satisfying Eqs (3) and (5). Points of intersections in the first quadrant are associated with the segment moving to the right (*c>0*), while the points of intersection in the third quadrant (*c<0*)–to the left. Model parameters used for plotting solid lines: *k*_*1*_ *= 2*.5*10^*−4*^, *k*_*2*_ = 2*k_*1*_, *D = 0*.*5*; *a = 50*, *c*_*0*_ *= -12*,*-8*,*-4*,*-1—*for (highest to lowest in the II quadrant) dotted lines and *c*_*0*_ *= 1*, *4*, *8 and 12*—for (lowest to highest in the I quadrant) solid lines. **B**: *u*-profile for the segment moving to the right (given by the Eq (3) where *c = 0*.*02*). **C**: *u*-profile for the segment moving to the left (given by the Eq (3) where *c = -0*.*02*).

Other roots of Eq (3) can’t be found analytically. However one can show that *f(c)→0* as *c→*∞ and therefore at least one non-zero root should exist if *f’(0)>1*. Indeed, the roots of Eq (3) are given by the points of intersection of curves *y = c* and *y = f(c)* and, therefore, if *f(c)>c* for small *c* (when *c>0*) (as follows from *f’(0)>*1) and *f(c)<c* for large *c* (as follows from *f(∞) = 0*) then there should be at least one point of intersection of these curves at some positive value of *c*. Also one can show that *f(c)* is an odd function and therefore roots of Eq (3) occur in pairs with the positive root giving the velocity of domain moving to the right and the negative root—moving to the left. These statements are illustrated by Fig 3A where plots of *f(c)* for four positive (dotted lines) and four negative (solid lines) values of parameter *c*_*0*_ together with the plot of *y = c* (dashed line) are given. This graph illustrates that the point of intersection between the solid and dashed lines corresponding to *c = 0* exists for all values of *c*_*0*_ while a pair of extra points of intersection appears only when *c*_*0*_ is negative and below a certain value.

Inspection of plots presented in Fig 3A indicates that nontrivial (*c≠0*) points of intersection of solid lines with the dashed line occur only when solid lines at *c = 0* raise quicker than the dashed line, that is when *df/dc(0)>1*. The transition point when the travelling domains (*c≠0*) satisfying Eqs (3) and (5) occur is given by the condition *df/dc(0) = 1* or, as it follows from Eq (5):

$$\frac{df}{dc}{|}_{c=0}=\frac{c_{0}k_{2}}{2ak_{1}\sqrt{Dk_{1}}}\left[e^{-a\sqrt{\frac{k_{1}}{D}}}\left(a\sqrt{\frac{k_{1}}{d}}+1\right)-1\right]>1.$$ (6)

The expression in the square brackets is always negative (for positive values of the involved model parameters) and therefore the formula confirms (as can be noted from Fig 3A) that *df/dc* is a decreasing function of *c*_*0*_ and *df/dc(0) = 1* for a certain negative value of *c*_*0*_, which we will denote as *c*_0_*:

$$c_{0}^{*}=\frac{2ak_{1}\sqrt{Dk_{1}}}{k_{2}\left[e^{-a\sqrt{\frac{k_{1}}{D}}}\left(a\sqrt{\frac{k_{1}}{D}}+1\right)-1\right]}\text{.}$$ (7)

**Fig 4:**
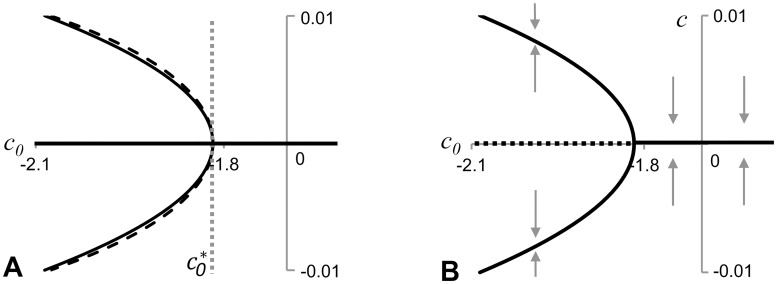
Plots illustrating dependence of the velocity of traveling wave solutions, *c*, in the system (2, 4), on the strength of chemotaxis, *c*_*0*_, and indicating the presence of a supercritical pitchfork bifurcation in Eq (8). **A**: Plots of *c(c*_*0*_*)* satisfying the system (3, 5) for analytical solution (solid black line) and its cubic approximation (dashed line) are shown (for the values of model parameters taken from Fig 3). Plots illustrate that non trivial travelling wave solutions exist below a critical value, *c*_0_*, of the bifurcation parameter *c*_*0*_. Travelling solutions emerge in pairs, moving with an equal speed in the opposite directions. **B**: Our stability analysis indicates that the pitchfork bifurcation is supercritical and solutions corresponding to travelling waves are stable.

This is a bifurcation value of the parameter *c*_*0*_: the system (3, 5) has only one solution when *c*_*0*_*>c*_0_* and three when *c*_*0*_*<c*_0_*. Plots given in Fig 4A indicate how the velocity of the travelling domain depends on *c*_*0*_. A solution represented by a stationary domain (*c = 0*) exists for all possible values of model parameters, while two extra solutions (travelling to the right and to the left) appear only when *c*_*0*_*<c*_0_*. Using cubic approximation of the function *f(c)* around *c = 0* one can show that the functions ±*c(c*_*0*_*)* describing these two extra solutions at the point of bifurcation (where they emerge from *c = 0*) can be represented by a hyperbola (dashed line in Fig 4A).

### Stability of cellular migration pattern in the basic model

Our next task is to analyse whether the cellular migration and corresponding travelling wave solution obtained in the basic model are stable. This analysis can be based on the study of stationary solutions in the system (2, 4) presented by the plot in Fig 2D. Let us consider the initial value Problem (2) where the function *u*_*0*_*(ξ)* is represented by a small deviation of one of the travelling wave solutions given by Eqs (3) and (5). Let us assume that while the *u*-profile evolves, the speed of the MD as given by the Eq (4) changes monotonically. Then we can state that the rate of change, *dc/dt*, is uniquely defined by the value of speed, *c*, and therefore there is a function *F(c)* such that *dc/dt = F(c)*. Furthermore, *dc/dt = 0* if *c = f(c)* (i.e. satisfies Eq (5)) and therefore the first order approximation of function *F(c)* is: *F(c) = α(c-f(c))* where *α* is unknown constant. Thus:

$$\frac{dc}{dt}=\alpha (c-f(c))$$ (8)

and stability of a travelling wave solution (say, moving with speed *ĉ*) depends on the sign of the derivative of the right-hand-side of Eq (8) *F'(ĉ) = α(1-f'(ĉ))*: if this derivative is negative the travelling wave solution (moving with velocity *ĉ*) is stable and otherwise—unstable. To conclude about stability of travelling solutions we only need to find the sign of constant *α*. It is intuitively clear that a non-moving domain (i.e. travelling wave solution corresponding to *ĉ = 0*) is stable in the case of chemoattraction, i.e. when *c*_*0*_*>0*. Plots in Fig 3A indicate that *f'(0)<1* when *c*_*0*_*>*0 and therefore *F'(ĉ) = α(1-f'(ĉ))<*0 (condition for the non-moving domain to be stable) if *α<0*. Nontrivial travelling wave solutions appear when *f’(0)>1*, that is when non-moving domain becomes unstable. Thus, the pitchfork bifurcation in Fig 3A should correspond to the supercritical case: travelling wave solution with *ĉ = 0* is stable when *c*_*0*_*>c*_0_* and unstable when *c*_*0*_*<c*_0_*. Two travelling wave solutions corresponding to moving domains appear when *c*_*0*_*<c*_0_* and since the bifurcation is supercritical they are stable. Their stability is confirmed by numerical simulations (S3 Movie) and by the observation from Fig 3A that *f'(ĉ)<1* where *ĉ* is the abscissa of non-trivial points of intersection of solid lines with a dashed line and therefore *F'(ĉ) = α(1-f'(ĉ))<*0 for nontrivial travelling wave solutions.

In the remaining part of this section (Eqs (9)–(15)) we provide a more formal analysis of the stability of travelling wave solutions which can safely be omitted by readers. This analysis is based on the study of the chemical profile dynamics in response to a small perturbation *δu(ξ*,*0)*. The perturbed *u*-profile satisfies Eq (2) although now the velocity *c* of the MD is not constant and:

$$\frac{dc}{dt}=\frac{c_{0}}{a}\frac{d}{dt}(u(a,t)-u(0,t)).$$ (9)

Thus, the evolution of the perturbed profile *u(ξ*,*t)* in a frame of reference moving with the instantaneous speed of the MD is described by the equation:

$$u(\xi ,t)=\delta u(\xi ,t)+\tilde{u}(\xi ,t)$$ (10)

where *δμ*(*ξ*, 0) is the original perturbation and *ũ*(*ξ*, 0) = *u*_*st*_(*ξ*, *ĉ*) is the unperturbed profile moving with the stationary speed, *ĉ*. The instantaneous velocity of the MD is given as:

$$c(t)=\hat{c}+\delta c(t)=\hat{c}+\frac{c_{0}}{a}[(\delta u(a,t)+\tilde{u}(a,t)-u_{st}(a,\hat{c}))-(\delta u(0,t)+\tilde{u}(0,t)-u_{st}(0,\hat{c}))],$$ (11)

The equation for the perturbation, *δu(ξ*,*t)*) is homogeneous (i.e. Eq (2) without term p(ξ)) and therefore it tends to zero. On the other hand the profile, *ũ(ξ*,*t)*, tends to the instantaneous steady state solution, *u*_*st*_*(ξ*, *c(t))*, given by Eq (3) where the instantaneous speed *c(t)* is given by Eq (11). Our task is to find out how *ũ(ξ*,*t)* evolves. It is easy to see that at time *t = 0*:

$$\begin{array}{l} \tilde{u}(\xi ,0)-u_{st}(\xi ,c(t=0))=u_{st}(\xi ,\hat{c})-u_{st}(\xi ,\hat{c}+\delta c(t=0))=\\ -\frac{\partial u_{st}(\xi ,c)}{\partial c}{|}_{c=\hat{c}}\delta c(t=0)=-\frac{\partial u_{st}(\xi ,c)}{\partial c}{|}_{c=\hat{c}}\left[\frac{c_{0}}{a}\left(\delta u(a,0)-\delta u(0,0)\right)\right] \end{array}$$ (12)

and this relationship should hold at all times:

$$\tilde{u}(\xi ,t)-u_{st}(\xi ,c(t))=-\frac{c_{0}}{a}\frac{\partial u_{st}(\xi ,c)}{\partial c}{|}_{c=\hat{c}}\left(\delta u(a,t)-\delta u(0,t)\right)\text{.}$$ (13)

Thus, getting back to the Eq (9) we get:

$$\begin{array}{l} \frac{dc}{dt}=\frac{c_{0}}{a}\frac{d}{dt}(u(a,t)-u(0,t))=\frac{c_{0}}{a}\left[\frac{d}{dt}(\delta u(a,t)-\delta u(0,t))+\frac{d}{dt}(\tilde{u}(a,t)-\tilde{u}(0,t))\right]=\\ =\frac{c_{0}}{a}\frac{d}{dt}(\delta u(a,t)-\delta u(0,t))\left[1-\frac{c_{0}}{a}\frac{\partial }{\partial c}(u_{st}(a,\hat{c})-u_{st}(0,\hat{c}))\right]=\;\\ =\frac{c_{0}}{a}\frac{d}{dt}(\delta u(a,t)-\delta u(0,t))\left[1-\frac{\partial f(\hat{c})}{\partial c}\right]\text{.} \end{array}$$ (14)

If we assume that the perturbation, *δu(ξ*,*t)*, decays with the relaxation rate *k*_*1*_ (which is the exact case for perturbations satisfying the following two conditions: *δu*_*ξ*_*(0*,*t) = δu*_*ξ*_*(a*,*t)*, and *δu*_*ξξ*_*(0*,*t) = δu*_*ξξ*_*(a*,*t)* and for the slowest mode of perturbation in general case) then Eq (14) transforms into:

$$\frac{dc}{dt}=-k_{1}\left(\delta c-\frac{df(\hat{c})}{dc}\delta c\right)=\;-k_{1}\left(c-f(c)\right)$$ (15)

which is identical to Eq (8) and confirms that *α* in Eq (8) is negative.

### Migration of cells attracted by external signal

Let us modify the model and consider scenario when the morphogen is produced outside the MD. The dynamics of u-profile in a frame of reference moving with the MD is still described by Eq (2) but the production term is modified and given as:

$$p(\xi )=\{\begin{array}{c} 0\text{ for }0\leq \xi \leq a\\ k_{2}\text{ for }\xi <0\text{ and }\xi >a \end{array}\;.$$ (16)

**Fig 5:**
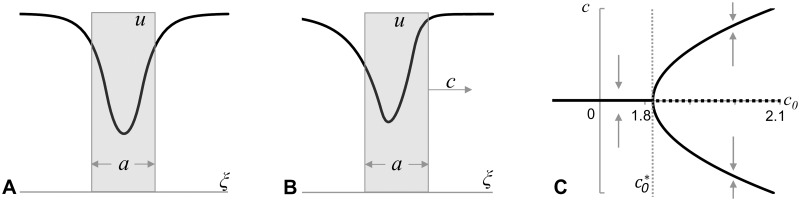
Travelling wave solutions and their stability in the systems (2, 16, 4). **A-B**: u-profiles for non-moving (**A**) and moving to the right (**B**) segments in the system (17, 18). **C:** Supercritical pitchfork bifurcation in Eq (8) for the system (2, 16, 4). Plots of analytical solutions *c = c(c*_*0*_*)* satisfying the system (17, 18) are shown. Model parameters are the same as in Fig 2, however now the production term in Eq (2) is given by Eq (16). When the chemoattraction gets strong enough, *c*_*0*_*>c*_0_*, the non-moving segment becomes unstable and two new (stable) solutions corresponding to moving segments emerge.

Stationary u-profiles in this model can also be found analytically:

$$u*(\xi ,c)=\frac{k_{2}}{k_{1}}-u_{st}(\xi ,c)$$ (17)

where *u*_*st*_*(ξ*,*c)* is given by Eq (3). Graphical representations of solutions corresponding to non-moving and moving to the right domains are shown in Fig 5A and 5B. Now we see that the MD advances towards higher concentration, that is, *u*-level is higher in the front of MD as compared to the *u*-level on its back. This indicates that the MD could be forced to move by a chemo-attraction mechanism, that is, when the cells forming the MD are attracted by the morphogen produced outside the MD. The velocity of the MD should satisfy Eq (4) and for the MD moving with a constant velocity (i.e. when *u(0)* and *u(a)* in Eq (4) are taken from Eq (17)) we will have

$$c=\frac{c_{0}k_{2}}{ak_{1}(\lambda_{1}-\lambda_{2})}\left(\lambda_{1}\left(e^{a\lambda_{2}}-1\right)+\lambda_{2}\left(e^{-a\lambda_{1}}-1\right)\right)\text{,}$$ (18)

which is identical to Eq (5) except for the inverted sign of the function on the right-hand-side. After following the same procedure as for the basic model and completing analysis of travelling wave solutions we conclude that nontrivial travelling wave solutions occur only when *c*_*0*_*>c*_0_* where *c*_0_* is a positive number (Fig 5C). The solution corresponding to a non-moving domain is stable if the chemotactic agent acts as a repellent (*c*_*0*_*<0*) or week attractor (*0<c*_*0*_*<c*_0_*). However when the chemo-attraction is sufficiently strong (*c*_*0*_*>c*_0_*) the solution corresponding to the non-moving domain becomes unstable while a pair of stable travelling (in opposite directions) wave solutions emerge. This result is a mirror translation of what was obtained for the basic model (Fig 3B): the stationary solution corresponding to non-moving domain becomes unstable under the sufficiently strong chemo-repulsion (*c*_*0*_*< c*_0_*<0) when the domain moves either to the right or to the left with the speed defined by the strengths of chemo-repulsion.

**Fig 6:**
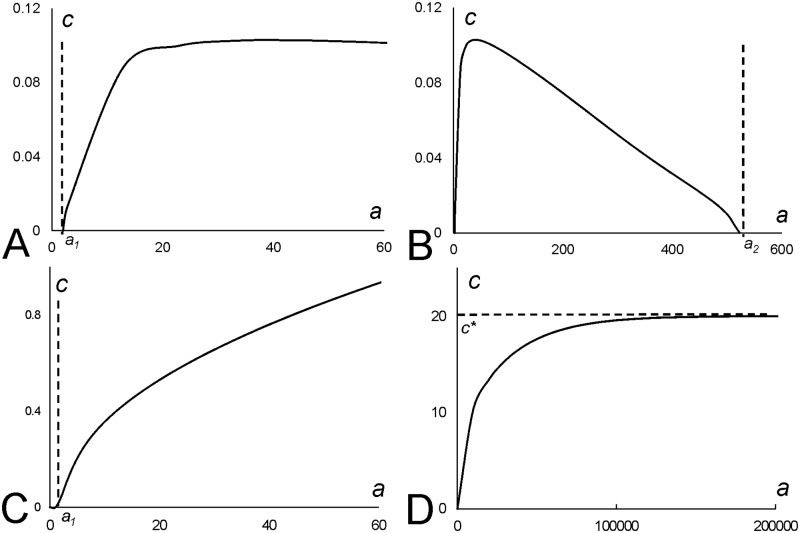
Speed of the MD versus its size in two modifications of the model. A-B: Solution of the Eq (5) for small- (A) and large-sized (B) segments. C-D: Solution of the Eq (22) for small- (C) and large-sized (D) segments. Plots are produced using MAPLE with the following values of model parameters: *k*_*1*_ *= 7*.5*10^*−4*^, *k*_*2*_ = 2*k_*1*_, *D = 0*.*5*, *c*_*0*_ *= 10*.

Eqs (5) and (18) for the velocity of chemotactic motion in response to internal and external signalling are almost identical and differ only by sign. This indicates that the speed of motion for these two scenarios depends on the model parameters in the same way. The speed monotonically increases with the product of parameters *c*_*0*_ and *k*_*2*_ (provided that this product is over a certain threshold) but has non-monotonic dependence on other model parameters including the size of the MD. Particularly, we have found that the speed increases with the size of the MD when this size is very small and decreases when the size is too large (see Fig 6A and 6B). The velocity has a maximum when the size of the MD is about the characteristic length ($l=\sqrt{D/k_{1}}$) in the concertation field of chemotactic agent. Besides the Eqs (5) and (18) imply that the domain can’t move if its size is below a certain threshold, $a_{1}\approx 2D\sqrt{Dk_{1}}/(\left|c_{0}\right|k_{2})$, or above the other threshold, $a_{2}\approx \left|c_{0}\right|k_{2}/(2k_{1}\sqrt{Dk_{1}})$, which are estimated using the approximations $e^{-a\sqrt{\frac{k_{1}}{D}}}=1-a\sqrt{\frac{k_{1}}{D}}$ for small *a* and $e^{-a\sqrt{\frac{k_{1}}{D}}}=0$ for large *a* in the inequality Eq (6).

### Passive migration due to chemotactic activity of surrounding cells

Now let us consider the scenario when the cells forming the MD are chemotactically passive while the cells in surrounding tissue respond chemotactically to the agent produced either by moving cells or by surrounding cells themselves. Let us denote the size of the tissue by *L* and the location of the MD by *x* (i.e. it occupies the segment [*x*, *x+a*]). Then according to the given above (bringing to the Eq (4)) arguments the front of the MD would have to move with the velocity $c_{x+a}=c_{0}\frac{u(L)-u(x+a)}{L-(x+a)}$ while its back with $c_{x}=c_{0}\frac{u(x)-u(0)}{x}$. Taking into account that the size of the MD is not changing we will have to set the same velocity to the front and to the back of the domain. Hewe we choose for the velocity of the MD to be equal to the sum of the above velocities for the front and the back (taking the arithmetic mean is another good option) that is: *c* = *c*_*x*+*a*_ + *c*_*x*_. As we see the domain can move even when it is formed by chemotactically passive cells however now its speed depends on its location and therefore will change over time. Assuming that the segment is located in the middle of the medium (*x≈L/2*) and the medium size is considerably larger than the characteristic length in the concentration field (so that *u(0)≈u(L)≈0*) we will have:

$$c\approx 2c_{0}\frac{u(x)-u(x+a)}{L-a}$$ (19)

Eq (19) is similar to the Eq (4) except for the opposite sign in the right-hand-side and the difference, *L-a*, replacing the size of the segment, *a*, in the denominator. Based on this similarity we conclude that the segment can move if cells in the surrounding tissue are:

1. either attracted by the agent produced inside the segment
2. or repelled by the agent produced by themselves.

As the medium size, *L*, appears in the denominator in Eq (19) the speed of segment decreases with the size of the medium. Besides, analysis of the solution of Eq (19) combined with the profile given by the Eq (2) indicates (not shown) that the domain can’t move if the medium size is over a certain threshold. The restriction that passive domain can’t move in a large medium comes from the way we model chemotactic response (adopted from the Koller-Segel model [19]), namely from the Eq (4). However there are a few ways to amend this equation which are justified by biological observations.

### Migration of cells under different assumptions concerning chemotactic forcing

It is feasible to assume that the cells respond to chemotactic signal only if the signal is strong enough, that is, if the gradient of chemotactic agent is above a certain value. As we see from the solution (3) the spike in *u*-concentration is located at the moving domain and the *u*-gradient is large enough only in the vicinity of the MD. We can estimate the size of this area by the characteristic length, $l=\sqrt{D/k_{1}}$, in the concentration field of chemotactic agent. This correction doesn’t make big difference when chemotactic activity is localised inside the MD (i.e. to formula (4)) however it allows significantly reduce the area of chemotactic activity in the case when chemotactic response comes from the surrounding tissue. Now we can reduce the size of chemotactically responding area from *L-(x+a)* in the front of the MD and from *x* in the back to $l=\sqrt{D/k_{1}}$ in both cases. This would allow modifying the Eq (19) in the following way:

$$c\approx c_{0}\frac{u(x)-u(x+a)}{\sqrt{D/k_{1}}}$$ (20)

Eq (20) allows movement of the domain composed by chemotactically passive cells in the arbitrary large medium and now the speed of the MD doesn’t depend on the medium size. Velocity defined by the Eq (20) is similar to the speed given by Eq (4) except for the characteristic length $l=\sqrt{D/k_{1}}$ replacing the size, *a*, of the MD in the denominator. This implies that if, for example, the characteristic length in *u*-concentration field is three-fold large than the size of the MD the passive migration defined by Eq (20) will be roughly three-times slower as compared with the speed of active migration defined by the Eq (4).

An alternative way to amend the Eq (4) is by adopting the concept that chemo-responding cells measure the difference of concentration over their length rather than the gradient in concentration filed [20]. Following this assumption we would need to modify the Eqs (4) and (5) so that the size of the MD is no longer in the denominators. Thus, the Eq (4) transforms into

$$c=c_{0}\left(u(a)-u(0)\right)$$ (21)

while the Eq (5) into:

$$c=\frac{c_{0}k_{2}}{k_{1}(\lambda_{1}-\lambda_{2})}\left(\lambda_{1}\left(1-e^{a\lambda_{2}}\right)+\lambda_{2}\left(1-e^{-a\lambda_{1}}\right)\right)\text{.}$$ (22)

The motion described by the Eq (22) is qualitatively different from that given by the Eq (5). Now the speed of the MD (in the infinite medium) monotonically increases (with saturation) when the MD size is increasing (see Fig 6C and 6D) while the speed of the MD described by the Eq (5) has a maximum at relatively small size of MD (see Fig 6A and 6B). In the case when the chemotactic activity is localised outside the MD (when the MD of size *a* moving in the medium of infinite size) we have for the velocity of its front *c*_*x*+*a*_ = −*c*_0_*u*(*a*) and for the velocity of its back *c*_*x*_ = *c*_0_*u*(0) with their sum giving the speed of the MD:

$$c=c_{0}\text{(}u(0)-u(a)\text{)}$$ (23)

which is a correction of the Eq (19). The Eqs (21) and (23) differ only by sign of their right-hand sides and therefore give the same speed for the MD. Thus, the MD moves with the same speed when the cells forming the MD produce repellent for themselves or attractant for surrounding cells, provided that all model parameters kept the same. Similarly, if we keep the values of model parameters unaltered but set the production area to be outside the MD then the domain will move and still have the same speed when the produced agent is attractor for the cells inside the MD or repellent for the cells outside the MD.

### Chemotaxis in two-dimensional tissue: Cellular Potts Model

So far we have been reducing the analysis of chemotactic motion of cells in a tissue by one-dimensional models. These models can easily be reformulated to analyse the process in two-dimensions and consider movement of a spot in planar tissue. However the analytical studies of two-dimensional models present certain challenges and therefore for this study we will use different modelling approach. Facing many problems which can’t be tackled analytically a community of mathematical biologists have developed a set of numerical models which are used to simulate biological processes. One of the most popular models currently used for simulation of cellular dynamics in tissue is called the Cellular Potts Model (or CPM, also known as HHGM) [21]. A detailed description of the model is given in a number of publications [9,18,22]. As a brief description we note here that the cells in this model are represented by a collection of grid points on a regular mesh while forces acting upon cells are considered indirectly by defining the Hamiltonian. In a basic model the Hamiltonian contains two terms which take into account the adhesiveness and incompressibility of cells. The dynamics of the system in this model is represented by Monte-Carlo process directed by the principle of minimization of free energy. Fig 2A is a snapshot of a model medium in basic CPM. To enable segregation of green and red cells it is assumed that the adhesiveness on the interface between green and red cells is weaker than between two green or two red cells. Definition of Hamiltonian is commonly extended by extra terms, particularly by one taking into account chemotactic properties of cells [9,22]. The energy change associated with this term refers to the work done by chemotactic force Δ*E*_*ch*_ ***= F***_*ch*_*∙****x*** and therefore corresponds to the chemotactic force, ***F***_*ch*_ = *β****grad****(u)*, exerted by chemotactically responding cell (*β = 0* for chemotactically passive cells, *β>0* for chemo-attraction and *β<0* –for chemo-repulsion).

**Fig 7:**
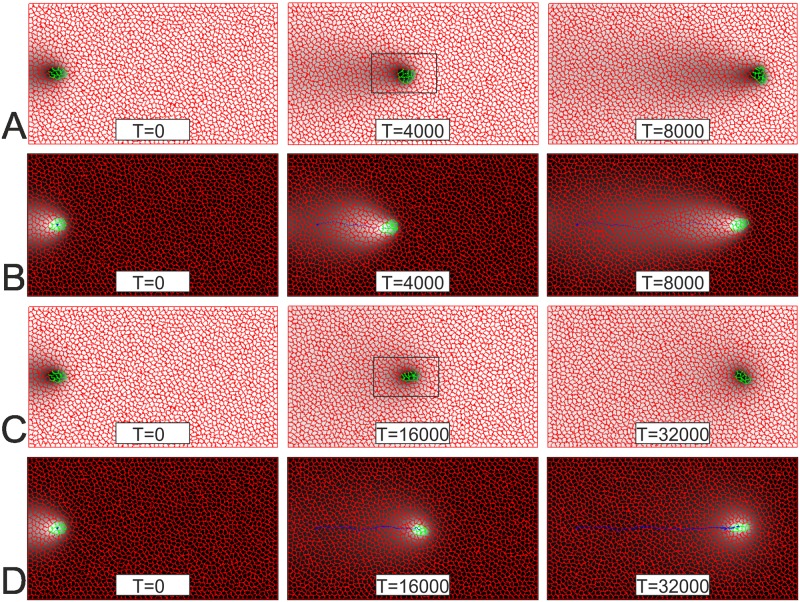
The MD in four chemotactic scenarios simulated using the Cellular Potts Model. **A:** Cells (green) forming the MD are repelled by the chemotactic agent produced by themselves. **B:** Cells (green) forming the MD are attracted by the chemotactic agent produced by surrounding (red) cells. **C:** Cells (red) surrounding the MD are attracted by the chemotactic agent produced by cells (green) forming the MD. **D:** Cells (red) surrounding the MD are repelled by the chemotactic agent produced by themselves. Locations of the MD at three consequent instances of time are shown for each scenario. Concentration of chemotactic agent is represented by shadows of grey from black (highest concentration) to white (lowest concentration). To maintain the direction of motion (to the right) the initial asymmetry was set in the following manner: green cells were initially set close to the left border; during initial 4000 time units the chemotaxis was turned off giving time for the concentration field to establish; then the concentration field was shifted 10 grid points left and chemotaxis was turned on. From this moment (*T = 0*) the domain was moving due to chemotaxis. The medium contains 31x55 cells, green domain– 9 cells, average cell contains 49 grids. Model parameters describing the dynamics of morphogen: *D = 0*.*5*, *k*_*1*_ *= 2*.5*10^*−4*^, *k*_*2*_ = 2*k_*1*_*; β = -8700*, both, time and space, steps are set to one. Other model parameters in CPM (for the explanation of these parameters see [9]) elasticity of cells in CPM, *λ = 0*.*6*; Boltzmann temperature, *T = 5*.*0*; adhesiveness matrix (cell_type1/cell_type2):$J=\left[\begin{array}{cc} red/red & red/green\\ green/red & green/green \end{array}\right]=\left[\begin{array}{cc} 3 & 7\\ 7 & 2 \end{array}\right]$.

In this part of our study we use the two-dimensional version of CPM to check the analytical results obtained on the basis of one-dimensional models. Particularly we confirm that all four described above mechanisms do indeed result in migration of the cellular domains in the CPM. The snapshots of simulated tissues in Fig 7 show the group of cells (shown in green) placed in a tissue composed by cells of another type (shown in red). Snapshots in Fig 7A illustrate the MD which is composed of green cells producing self-repellent. Snapshots in Fig 7B illustrate the MD composed of cells which are attracted by chemical produced by surrounding (red) cells. While the MDs in Fig 7A and 7B are formed by chemotactically active cells, Fig 7C and 7D illustrate passive migration of the domain, i.e. when cells forming the MD are not chemotactically active. Snapshots in Fig 7C are taken from the simulation when the chemical produced in the MD attracts surrounding cells, while in Fig 7D—from the simulation when surrounding cells produce self-repellent. All model parameters are identical in all four presented simulations. One can note that the speed of the domain migration is pretty much equal for both active mechanisms: the MDs in Fig 7A and 7B cover roughly the same distance in 8000 time units. Similarly the speed of the domain is the same for two passive mechanisms: in Fig 7C and 7D the MDs cross the simulated tissue in 32000 time units. Thus, in the framework of the CPM, active movement is four-times faster than the passive one.

The chemotactic forcing in Cellular Potts model is given by the parameter *β* which is similar (but not identical) to the parameter *c*_*0*_ in the considered above one-dimensional model. Besides the shape of the spot in CPM is not circular and exhibits considerable fluctuations following the underlying Monte-Carlo algorithm (see S4–S6 Movies). These fluctuations affect the average speed of migrating spot which is different from one simulation to another. This is also evident from comparing the snapshots in Fig 7A and 7B (or in Fig 7C and 7D) which are not identical although should be according to 1D-model. For example, the speed of the active MD (i.e. in the simulations shown in Fig 7A and 7B) is about 3.3 space units per 100 time units and varies in the range of ±6% between simulations with the same model parameters but different seeding of random numbers underlying Monte-Carlo process.

Important observation concerning the simulations shown in Fig 7 is about the direction of the domain migration. If we start simulation with unstable (i.e. apply a sufficiently strong chemotactic force) non-moving spot it eventually will start to move, however the direction of its motion appears to be random and can’t be predicted. For simulations shown in Fig 7 we have designed special initial conditions to ensure that the spot will migrate to the right. The group of green cells was initially placed next to the left border of the medium and set to move to the right by shifting the concentration field of the chemotactic agent to the left (with respect to the green group), This shift has introduced initial asymmetry in the model medium and allowed to control the direction of cell group migration. Initial asymmetry can be introduced by other ways, for example by starting with already moving domain. We have checked this by introducing artificial force acting on all green cells and pointing towards the right border of the medium. This force can be applied for a certain time (i.e. 2000 time units) and then be removed. Then for the rest of the simulation the group will keep moving to the right due to chemotaxis.

### Analysis of patterns formed by moving cells in Cellular Potts Model

The main assumption made in the 1D-model is that all cells forming the MD have the same velocity while all surrounding cells are immobile. This is certainly not the case for the MD in Cellular Potts Model where green cells forming the MD should make their pass across the area occupied by the red cells. Technically green cells move with different velocities and this is reflected by changes in the shape of the MD. However green cells move as a compact group (they are kept together due to differential adhesion) and in a long run (in average) can be considered as moving with equal velocities.

**Fig 8:**
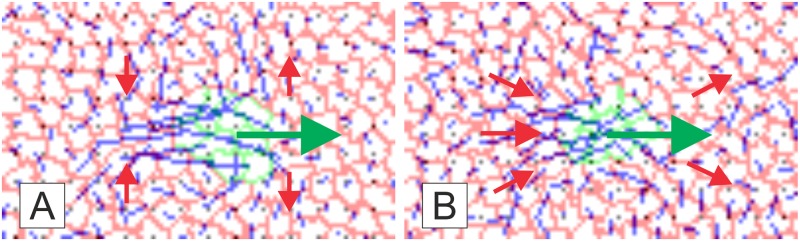
Movement patterns of cells in the vicinity of the MD in Cellular Potts Model. Panel **A** illustrates cell movement pattern for the two active mechanisms while panel B—for passive. Panel **A** shows the area framed in the snapshot for *T = 4000* in Fig 7A, while panel B—framed in Fig 7C, *T = 16000*. Both panels demonstrate locations of green and red cells and their traces, which are shown in blue and taken for 500 time units in panel A and 2000 time units in panel B (four-fold difference in time intervals compensates four-fold difference in speeds of the active and passive MDs). Conclusions on movement directions for (green and red) cells in different locations are illustrated by (green and red) arrows.

Analysis of the movement patterns for red cells, in simulations shown in Fig 7, indicates that the MD doesn’t affect behaviour of distant cells whose location only exhibit small fluctuations. However the red cells in the vicinity of the MD do indeed move and these movement patterns are pretty much identical for both active scenarios (i.e. for simulations shown in Fig 7A and 7B) and for both passive scenarios (for simulations shown in Fig 7C and 7D). But there is a significant difference between movement patterns of red cells depending on whether the migration takes place due to chemotactic activity of cells inside or outside of the MD (i.e. whether the MD is formed by chemotactically active or passive cells). Two panels in Fig 8 illustrate movement patterns of cells inside and in the vicinity of the MD in these two cases.

Fig 8A shows that while the green cells forming the MD move horizontally, the red cells mainly exhibit vertical motion. One can state that when the red cells are chemotactically passive they shift up-and-down to give space in the front of the actively moving domain and returning to their original position after the MD has passed. This observation fits the assumption used in 1D-model: immobility of surrounding cells in 1D-model is equivalent to the absence of horizontal motion for red cells in 2D-model. Fig 8B shows that when the MD formed by chemotactically passive cells and moves due to chemotactic activity of red cells the traces of red cells are mostly not vertical (although red cells just above and below the MD still move vertically) and have more profound horizontal component. Moreover, the red cells in the back of the MD move horizontally and seem to rather push the MD. This observation is in contradiction with the “immobility” assumption in 1D-model. Thus, based on the patterns of individual cell migration presented in Fig 8 we conclude that our analytical model should be better comparable with two-dimensional simulations of actively moving domain (absence of horizontal motion of red cells in Fig 8A corresponds to immobile surrounding) while horizontal component of red cells motion in the case of passive MD (Fig 8B) violates the assumption about immobility of surrounding cells.

Simulations show that the speed of the MD moving due to chemotactic activity of surrounding cells (Fig 7C and 7D) is roughly four-times slower than the speed of the MD composed of chemo-active cells (Fig 7A and 7B). As the description of chemotaxis in CPM is underlined by the Eq (4) we would expect that the passively moving domain (shown in Fig 7C and 7D) would move *a/(L-a)* times, that is 17 times (a = 3*7 = 21 and L = 55*7 = 385), slower than actively moving domains (shown in Fig 7A and 7B). This indicates a considerable numerical discrepancy between the simulations in the CPM and our analytics. Another important observation is that the speed of the passively moving cellular domain in the CPM doesn’t depend on the medium size. The last observation is in line with the model based on the modification of chemotactic force and described by the Eq (20) (the speed defined by this formula also doesn’t depend on the medium size) although the numerical discrepancy still persists: the ratio of speeds for the MDs moving under active and passive mechanisms is given by $a/\sqrt{D/k_{1}}$ which is 1/7 for the model parameters in Fig 7, while the simulations on the CPM give 1/4. This discrepancy can probably be explained by the fact that the assumption of immobility of surrounding cells underlying analytical model is violated in the case of passive motion (Fig 8B).

## Discussion

There is a long record of mathematical studies of chemotactic motion of cells in which however cells are either considered to be isolated and to move independently [19,23] or to be driven by periodic signals [24–26]. There is also a long record of experimental studies of cellular flows in tissues [11,15,27]. In this work we apply the mathematical formalism developed for the description of chemotactic motion of cells to the analysis of cellular dynamics in tissue. We have introduced a few modifications of one-dimensional continuous model designed for the study of motile dynamics of a single cell or group of cells in tissues. Based on the analysis of these models we derived conditions for the domain of cells to exhibit steady migration over tissue given that it is caused by chemotaxis. Two scenarios are associated with the case when the MD is formed by chemotactically active cells. That is, the group moves if it is either (sufficiently strongly) repelled by the chemical produced inside the group or attracted by the chemical produced outside the group. The first of these two mechanisms was observed experimentally [28] and was a subject in a number of theoretical studies, including analytical and numerical investigations of chemotactic motion of “sizeless” Brownian particles [12,23]. Particularly, this mechanism was used to explain the migration of stem zone during late gastrulation in the chick embryo [9]. The second mechanism (the motion due to attraction by surrounding cells) although looks obvious and would intuitively be expected has not previously been reported. This is probably because this mechanism can’t be in play in movement of isolated cells but can work when cells move in compact environment (i.e. tissues) build by other cells.

Two mechanisms of passive motion, when the domain moves due to chemotactic activity of surrounding cells, are neither would intuitively be expected nor previously reported. Our prediction of such migration is based on the analysis of one-dimensional models and we illustrated it numerically using the CPM (Fig 7C and 7D and also S6 Movie). Simulations on the CPM show that the speed of the passively moving domain doesn’t depend on the medium size and roughly four-times lower than the speed of the domain formed by chemo-active cells. This two observations form a discrepancy with one-dimensional models which we used to predict the phenomenon. The treatment of chemotaxis in the CPM is done according to the formula (4) which would rather mean that the speed of the MD should decrease with the size of the medium. The discrepancy can be explained by the observation that in the CPM cells surrounding the MD are moving too (Fig 8). This motion, which is neglected in the one-dimensional model, is probably a cause of the observed discrepancies; particularly why despite the fact that the chemotaxis is described by the Eq (4) the speed of the MD is not described by the Eq (19) and doesn’t depend on the size of the tissue.

Two mechanisms of active motion of cells are mathematically identical and this is reflected by the fact that the speeds of the MDs are equal in both scenarios provided that the values of model parameters are the same. Similar statement holds for two passive mechanisms. Evidently active mechanisms are more efficient in a sense that in systems with qualitatively similar chemical and taxis dynamics active mechanisms result to considerably faster migration of cells. Besides, to the best of our knowledge, no observations indicating that either of passive mechanisms is in play have been published so far. Contrary to this, at least one of the mechanisms of active motion, that is the phenomenon of self-repulsion is well known and have been observed in biological [28] and physical [29,30] systems. Active motion due to self-repulsion has certain advantages as compared to the one due to attraction by surrounding cells: it is self-contained as it doesn’t rely on the environmental conditions and more efficient in terms of the amount of produced chemical. Thus self-repulsion seem to be a reasonable mechanism to be involved in migration of cells in tightly regulated processes such as embryogenesis. Contrary to this, migration due to attraction by surrounding cells might be in play in misregulated processes such as spreading of metastasis. Indeed, the phenomenon known as haptotaxis (cellular migration along the gradient of adhesive molecules [31]) can be viewed as an example of the case when cells migrate due to attraction by surrounding cells.

One can also note a large difference in absolute values of parameters *c*_*0*_ (in the one-dimensional model) and *β* (in the CPM) which allow migration of domains due to chemotaxis: for example, the value of *β* used in the simulations shown in Fig 7 is by almost three orders of magnitude higher than the value of *c*_*0*_ used for plots in Fig 6. To explain this discrepancy we note that *c*_*0*_ defines the speed of motion while *β*–the force exerted by chemo-active cells. These two descriptions are linked by the equation of balance of forces: ***F***_*ch*_+ ***F***_*fr*_ *= 0*, where ***F***_*ch*_ = *β****grad****(u)*–force exerted by chemo-active cells and ***F***_*fr*_ = *-k*_*fr*_***c***—friction to the motion with velocity ***c***. It follows that

$$c=\frac{\beta }{k_{fr}}grad\text{(}u)\;=c_{0}grad\text{(}u\text{)}$$

indicating that stronger resistance to cell motion results to larger difference between the bifurcation values of parameters *c*_*0*_ and *β*. Particularly when the MD is isolated and not making its pass through the tissue it doesn’t experience strong resistance force and the value of *β* allowing such migration is considerably lower [9].

Model parameters can be scaled in an assumption that the simulations shown in Fig 7, where the size of moving group is 20 space units and it covers 300 space units in 8000 time units, describes the movement of the stem zone (Fig 1C) which has a size of 0.3 mm and moves with a speed of 2μm/min. Then the space unit in the model should correspond to 15μm and time unit to 18 sec. This means that *D = 0*.*5* corresponds to the diffusion constant of 6 μm^2^/sec and *k*_*1*_ *= 2*.5*10^*−4*^ corresponds to the decay rate of 10^−5^ sec. Both these values are in the range of experimental measurements made for a morphogen (Lefty) in zebra fish embryo [32] which is, similarly to FGF8, represented by a small protein. Also, the characteristic length in the concentration field of chemotactic agent (in the simulation shown in Fig 7) is $l=\sqrt{D/k_{1}}=100/\sqrt{5}\approx 45$ space units or 670 μm. Assuming that u = 1 corresponds to micro-molar concentration and taking the size of a cell to be around 10 μm we conclude that the difference in *u*-concentration across the cell can be estimated as 10 nano-molar and cells are known to be able to detect this difference [20].

The presented work can be extended in a few directions. In real tissues there are more than two cell types and this brings to an interesting problem of migration of non-uniform group of cells [8]. That is, the moving group may be composed of two sub-populations, of which one is chemo-active, while the other is not. Furthermore, in embryonic tissues moving cells proliferate and differentiate and this leads to more sophisticated scenarios when the same chemicals are responsible not only for motion of cells but also for their differentiation [9]. Finally, a number of studies indicate that the migration of cells in tissues can be due to cellular interaction [11] rather than chemotaxis and for consideration of this scenario completely different models should be developed.

## Supporting Information

S1 Movie Moving domain in a laboratory frame of reference. The speed of motion, *c = 0*.*02*, is pre-set, the segment is initially located at the left border, *u*-profile is calculated according to the Eq (1). Model parameters: *k*_*1*_ *= 2*.5*10^*−4*^, *k*_*2*_ = 2*k_*1*_, *D = 0*.*5*; *a = 50*; *u(x*,*t = 0) = 0*.
(MPEG)

S2 Movie Moving domain in a co-moving frame of reference. The speed of motion, *c = 0*.*02*, is pre-set, *u*-profile is calculated according to the Eq (2). Model parameters: *k*_*1*_ *= 2*.5*10^*−4*^, *k*_*2*_ = 2*k_*1*_, *D = 0*.*5*; *a = 50*; *u(x*, *t = 0) = 0*.
(MPEG)

S3 Movie Movie demonstrating the instability of the solution with *c = 0*. Simulations are performed in a laboratory frame of reference (according to the Eq 1) with the speed, *c*, calculated according to the Eq (4). Model parameters: *k*_*1*_ *= 2*.5*10^*−4*^, *k*_*2*_ = 2*k_*1*_, *D = 0*.*5*; *a = 50*, *c*_*o*_ *= 2*, *u(x*, *t = 0) = 0*. At time step t = 40000 the domain is shifted one grid point to the left and this results to the transformation of the solution from non-moving to travelling segment.
(MPEG)

S4 Movie Motion of the group of cells in the 2D-tissue simulated by CPM. Group containing 9 cells (represented by the spot framed with double green/red contour) produces the chemical (concentration is indicated by shadows of grey) which acts as a chemorepellent to these cells. As a result of chemo-repulsion this group of cells migrates through the tissue formed by other cells. To maintain the direction of motion we have introduced initial asymmetry in the following way: the group of green cells was initially placed next to the left border of the medium and left unperturbed for 4000 time steps to allow production of the chemical. Then the concentration field of the chemical was slightly (by 5 grid points) shifted to the left and chemo-repulsion was switched on. For the rest of the simulation the group was moving due to chemotaxis. For model parameters see the Fig 7 legend. Boundaries between individual cells are not shown.
(MPEG)

S5 Movie Motion of the group of cells in the 2D-tissue simulated by CPM. Group containing 9 cells is attracted by the chemical (concentration is indicated by shadows of grey) produced by the surrounding cells. As a result of chemo-attraction the group of cells migrates through the tissue formed by other cells. For model parameters see the Fig 7 legend. Boundaries between individual cells are not shown.
(MPEG)

S6 Movie Motion of the group of cells in the 2D-tissue simulated by CPM. Group containing 9 cells (shown in green) produces the chemical (concentration is indicated by shadows of grey on the right panel) which attracts surrounding cells (shown in red). As a result of this chemo-attraction the group of cells migrates through the tissue formed by other cells. For model parameters see the Fig 7 legend.
(MPEG)
